# A Case of Synovial Chondromatosis of Temporomandibular Joint with Numerous Loose Bodies

**DOI:** 10.1155/2021/5927215

**Published:** 2021-12-11

**Authors:** Yumiko Matsusue, Kazuhiko Yamamoto, Nobuhiro Yamakawa, Ikumi Yamamoto, Shinpei Matsuda, Hitoshi Yoshimura, Tetsuji Kawakami, Tadaaki Kirita

**Affiliations:** ^1^Department of Oral and Maxillofacial Surgery, Nara Medical University, Japan; ^2^Department of Oral and Maxillofacial Surgery, Takita Hospital, Japan; ^3^Department of Dentistry and Oral Surgery, Unit of Sensory and Locomotor Medicine, Division of Medicine, Faculty of Medical Science, University of Fukui, Japan

## Abstract

Here, we report a case of synovial chondromatosis of the temporomandibular joint (TMJ) with numerous loose bodies. A 56-year-old woman was examined in the oral surgery department for trismus and pain in the left TMJ when opening the mouth. Imaging indicated TMJ synovial chondromatosis, and the patient was referred to our department for further examination. Her facial features were symmetrical, and no occlusal abnormalities were found. The maximum mouth opening was 30 mm, and movement of the left mandibular condyle was restricted and accompanied by pain and joint sounds. Panoramic radiography showed deformation of the left mandibular condyle and radiopaque lesions surrounding it. Computed tomography showed numerous small granules around the left mandibular condyle, some of which were calcified. Magnetic resonance imaging showed anterior disc displacement without reduction in the left TMJ and hypointense lesions on T2-weighted images. Bone scintigraphy showed an accumulation in the area of the left TMJ. Based on the diagnosis of the left TMJ synovial chondromatosis, the lesions were removed, and plastic surgery on the mandibular condyle was performed under general anesthesia. We removed 386 white loose bodies. Histopathologically, the loose bodies were consistent with synovial chondromatosis lesions. The postoperative course was uneventful, with no recurrence or TMJ dysfunction approximately 5 years after the surgery, indicating that open surgery is the best course of intervention in such cases.

## 1. Introduction

Synovial chondromatosis is a benign disease in which synovial cells create cartilaginous metaplasia in synovial tissue, resulting in loose cartilage bodies in the joint cavity. The most common sites include the knee, elbow, and hip. Synovial chondromatosis of the temporomandibular joint (TMJ) was first described by Von Haller in 1764 [1]. Synovial chondromatosis is not frequently encountered in the TMJ, but reports of such cases have increased along with recent advances in diagnostic imaging, including magnetic resonance imaging (MRI). Here, we report a case of TMJ synovial chondromatosis with numerous loose bodies to draw attention to this rare condition and suggest a suitable course of action for its treatment. We also review the related literature of reported TMJ synovial chondromatosis cases.

## 2. Case Presentation

A 56-year-old woman presented to our department with a chief complaint of pain in her left TMJ in December 2014. Around November 2012, the patient had noticed pain in her left TMJ when widely opening her mouth, but the symptoms disappeared, and she did not address the matter. Around July 2014, the symptoms recurred, and she began experiencing trismus. She visited the dental and oral surgery department at a hospital for examination. Both computed tomography (CT) and MRI indicated synovial chondromatosis of the TMJ, and she was referred to our department for treatment.

**Figure 1:**
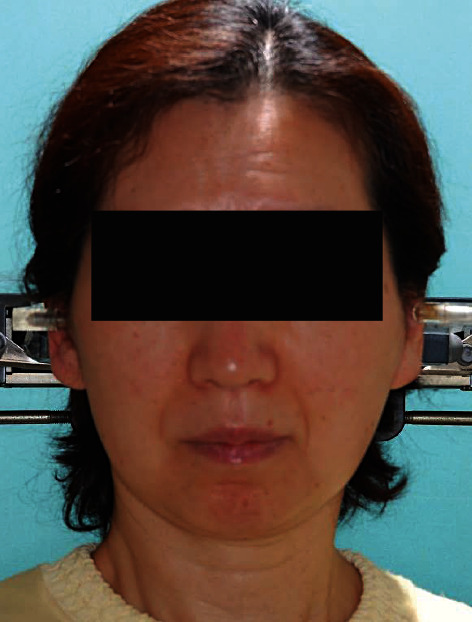
Facial photograph from the first examination. There is no visible facial deformation.

Clinical examination found that she had a fairly healthy physique and no signs of nutritional deficiency. There was no visible facial deformation, and the maximum mouth opening was 30 mm. Movement of the left mandibular condyle was poor, and there was pain as well as joint sounds when widely opening the mouth but no occlusal abnormality (Figure 1).

**Figure 2:**
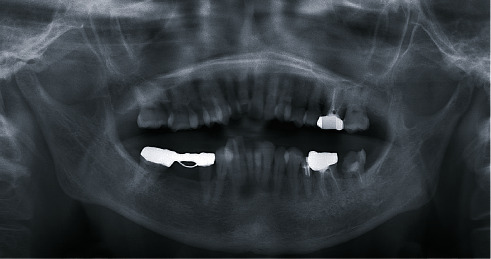
Panoramic radiograph from the first examination. The left mandibular condyle is flattened, and radiopaque images are seen near the superior joint cavity.

**Figure 3:**
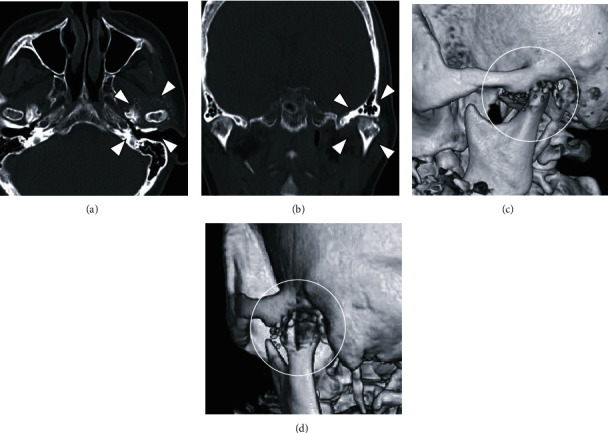
CT images from first examination: (a) axial image, (b) coronal image, and (c, d) 3D image. Deformation of the left mandibular condyle is seen, with many small, granular nodules in the area of the left TMJ, some of which are calcified.

Panoramic radiography showed that the left mandibular condyle was flattened, with a radiopaque image near the superior joint cavity (Figure 2). The TMJ radiograph also showed deformation in the left mandibular fossa. With mouth opening, both mandibular condyles were able to move to near the lowest point of the articular eminence. CT showed that the left mandibular condyle was flattened, and small and granular nodules were observed in the joint capsule of the left TMJ, some of which were calcified (Figure 3).

**Figure 4:**
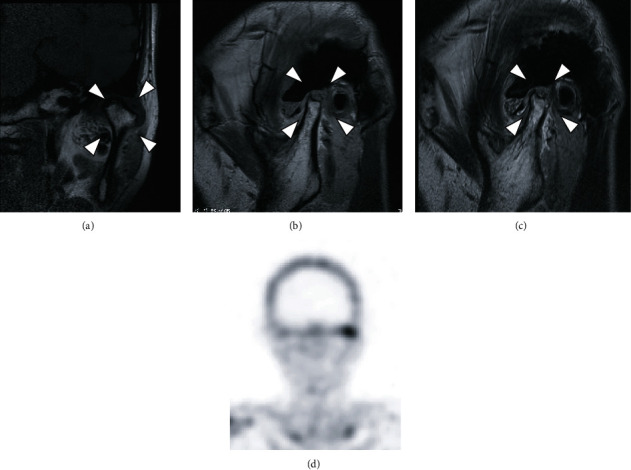
MR image and bone scintigraphy from the first examination. (a, b) T1-weighted image; (c) T2-weighted image. In the left TMJ, T1-weighted images show deformity of the left mandibular condyle and anterior disc displacement without reduction, and T2-weighted images show areas of small, nodular hypointensity around the left mandibular condyle. (d) Bone scintigraphy. An accumulation is seen in the area of the left TMJ.

T1-weighted MRI of the left TMJ showed deformity of the left mandibular condyle and anterior disc displacement without reduction, and T2-weighted MRI showed fine nodular areas of hypointensity in the superior joint cavity (Figures 4(a)–4(c)). Bone scintigraphy showed accumulation at an area matching the left TMJ (Figure 4(d)). Based on these findings, the final diagnosis was of left synovial chondromatosis of the TMJ.

Approximately 2 months after the initial examination, the masses were removed, and plastic surgery was performed on the mandibular condyle under general anesthesia. Incision lines were set according to the Al-Kayat–Bramley method [2]. The skin incisions were made, detaching up to the temporal fascia. An incision was made in the superficial temporal fascia, then in between the superficial and deep layers to the zygomatic arch, where the periosteum was incised to expose the joint capsule. The joint capsule was punctured, and approximately 2 mL of slightly viscous synovial fluid was collected. Incising the joint capsule revealed numerous white loose bodies around the mandibular condyle and in the superior joint cavity. The loose bodies were not adherent to the surrounding tissue and were carefully removed.

**Figure 5:**
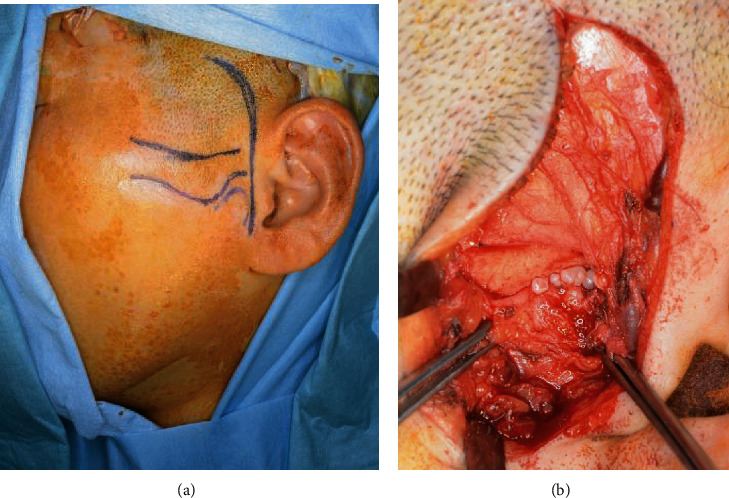
Intraoperative findings: (a) skin incision line (arrowhead); (b) numerous white loose bodies exposed after incision of the joint capsule.

Although the articular disc was preserved, the mandibular condyle was deformed. Therefore, plastic surgery of the mandibular condyle was performed using an ultrasonic cutting instrument (Piezosurgery®, Mectron Medical Technology, Carasco, Italy). A closed drain was placed in the wound, and the incision was sutured to complete the operation (Figure 5).

**Figure 6:**
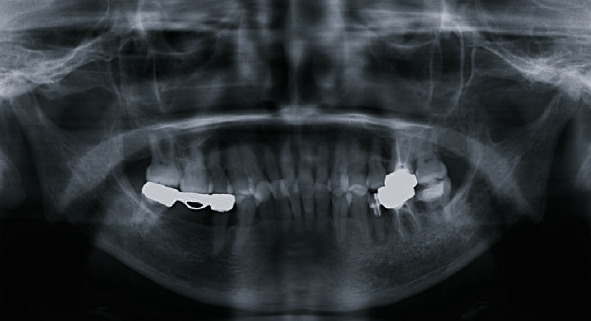
Panoramic radiograph approximately 2 years after the surgery. There are no findings suggesting recurrence.

**Figure 7:**
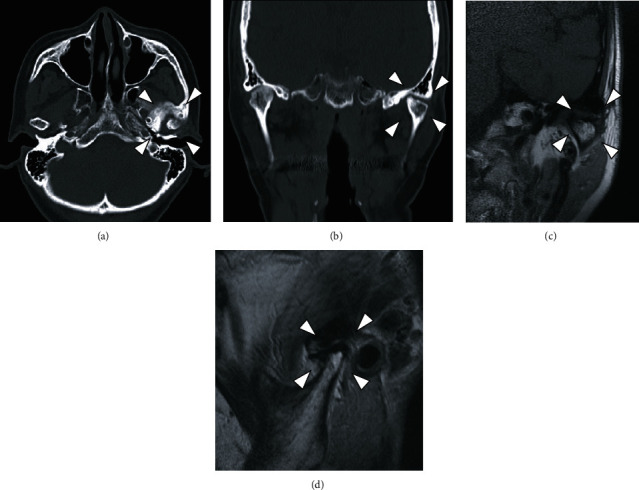
CT and MR images approximately 2 years after the surgery: (a, b) CT images—(a) axial image and (b) coronal image; (c, d) MR images, T1-weighted image. None of the images suggest recurrence.

At the time of writing, it has been more than 5 years since the surgery. There have been no signs of local recurrence or trismus. Image findings have also not indicated any recurrence (Figures 6 and 7).

**Figure 8:**
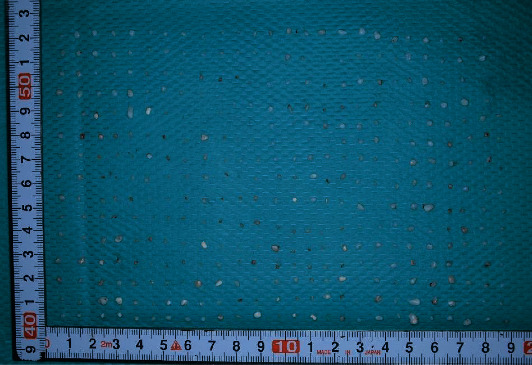
Extracted specimens. A total of 386 white, bone-like, hard, loose bodies of 2–5 mm in diameter were removed.

The loose bodies removed were 2 to 5 mm in diameter, white, bone-like, hard, and numbered 386 in total (Figure 8).

**Figure 9:**
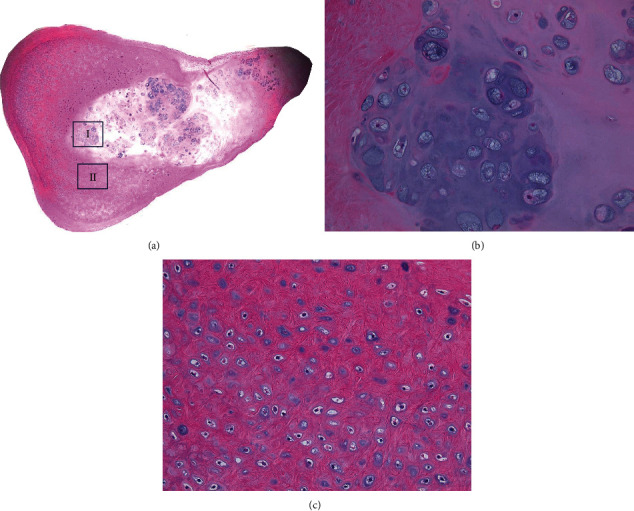
Histopathological findings: (a) hematoxylin-eosin staining ×1.25, low magnification; (b) hematoxylin-eosin staining ×200, high magnification of AI; (c) hematoxylin-eosin staining ×200, high magnification of AII. Cartilage tissue with growth of chondrocytes forming round or irregular small masses surrounded by fibrous tissue. Fibrosis and ossification are observed in some areas.

Histopathologically, there was proliferation of cartilage tissue with chondrocytes forming round or irregular small masses surrounded by fibrous tissue, along with fibrosis and ossification. There were no signs of malignancy, which is consistent with synovial chondromatosis (Figure 9).

## 3. Discussion

In this report, we presented the case of a patient with TMJ synovial chondromatosis with numerous loose bodies who was successfully treated with surgery and has remained symptom free 5 years after the operation. Synovial chondromatosis of the temporomandibular joint presents with clinical symptoms similar to TMJ disorder, including trismus, restricted movement of the TMJ, pain, and noise [3, 4]. Thus, patients are sometimes treated for TMJ disorder for an extended period rather than receiving proper treatment [5]. Imaging is essential for diagnosing this disease, but simple radiography often does not show any changes. CT depicts joint and soft tissue swelling, multiple calcifications in a joint, or hardening and resorption of joint surfaces, but lesions may not be depicted in the absence of obvious calcification or bone changes. MRI shows enlargement of the joint cavity, synovial fluid retention, and a swollen joint capsule with multiple hypointense masses in T2-weighted images [6–8]. Diagnosis is relatively easy in cases with numerous calcifications, such as the present one, but when these are not visible, diagnosis can be complicated. Therefore, both CT and MRI should be used to ensure a comprehensive diagnosis incorporating all clinical findings [9–11]. This condition must be differentiated from other possible diagnoses such as TMJ internal derangement, tuberculous arthritis, osteochondrosis ossificans, osteochondral fracture, and pseudogout [12]. If accompanied by intracranial progression [13], conditions such as meningioma, schwannoma, chondrosarcoma, lymphoma, and cholesteatoma should be considered [4].

Milgram [14, 15] classified synovial chondromatosis of the knee, elbow, and hip into three stages and considered it a self-limited disease that progresses from stages 1 to 3 in this order. In stage 3, synovectomy is not necessary if the loose bodies are removed, and even in stage 2, synovectomy is not always required. Gerard et al. [16] classified the disease into four stages of histopathological changes in synovial activity. They recommend expansion surgery in the early stages and reduction surgery in the late stages of synovial chondromatosis. Our case presented as stage 3 according to Milgram's classification and stage 2 according to Gerard's classification. By carefully removing as many of the numerous loose bodies as possible and performing plastic surgery of the mandibular condyle, we minimized postoperative dysfunction, indicating that this intervention was appropriate.

We located 202 case reports of TMJ synovial chondromatosis, including the present one, published from 2009 to 2019 [7, 11, 17–58] (Table 1). The cases covered a wide age range from 15 to 86 years and included 144 women and 58 men. The clinical symptoms were swelling in 60 cases, pain in 104 cases, trismus in 97 cases, and joint sounds in 67 cases. All patients were examined at a medical institution for these symptoms. Approximately 90% of patients had open surgeries, but approximately 10% had arthroscopic surgeries. In addition to removing the lesions, resection arthroplasty was performed in 14 cases, mandibular plastic surgery or resection in 8 cases, discectomy in 11 cases, and synovectomy in 12 cases. Conservative surgery was selected if the surrounding tissue was normal. Loose bodies were found in most cases, but ours was the only one where more than 300 loose bodies were removed.

The therapy for TMJ synovial chondromatosis is principally surgical intervention, as the number of loose bodies increases or the symptoms worsen [59]. If the loose bodies are confined to the superior joint space, arthroscopic surgery can be selected, although some studies have reported recurrence after arthroscopic surgery [60]. If the goal is definite cure, we believe that open surgical resection is the best option.

Synovial chondromatosis is characterized by multiple chondrodysplasia or osteochondrodysplasia and free body formation in a joint due to synovial metaplasia, but its underlying pathological mechanism remains unclear. Possible triggers are trauma and continuous irritation to the synovial membrane [61]. Trauma is considered to lead to the production of cartilage from secondary synovial changes, while continuous stimulation may trigger cartilage formation from synovial cell metaplasia due to internal joint damage. However, our patient had no history of trauma. We believe that anterior disc displacement allows rich connective tissue growth in the posterior synovium, which is chronically stimulated by the mandibular condyle. Although the molecular mechanism of this disease is not understood, immunohistochemical studies of loose bodies from synovial chondromatosis have found expression of fibroblast growth factor-2 and fibroblast growth factor receptor-1 in chondrocytes, suggesting their involvement in the pathology [62, 63]. In a study of synovial tissue from TMJ synovitis, interleukin-1*β* and tumor necrosis factor *α* were expressed in both the synovial cell layer and the vascular wall [64, 65]. These reports indicate that the onset of this disease is influenced by a mechanism involving inflammatory cytokines and other substances produced by the synovial membrane or microvascular disorders that regulate the growth and activity of synovial cells against a background of genetic or environmental factors [66, 67]. However, in addition to the above factors, various cytokines, growth factors, pain-producing substances, and other substances involved in inflammation and cell proliferation are known to be expressed in the synovial fluid and synovial cells of the TMJ [65, 68]. Clarifying the locations and effects of these substances could help provide diagnostic indicators for the diagnosis of this type of synovial chondritis and its pathological progression.

The prognosis of this disease is relatively good, and recurrence is uncommon. However, because of reports of malignant transformation to chondrosarcoma in other joints [6, 69, 70], we plan to continue to follow-up this patient.

In conclusion, we reported a rare case of TMJ synovial chondromatosis with 386 loose bodies, which were removed using open surgery. There has been no recurrence in the 5-year follow-up period, indicating that open surgery is the best course of intervention in such cases.

## Figures and Tables

**Table 1:** Summary of data from the studies included in the review, 2009 through 2019.

No.	First author	Year	No. of cases	Age	Sex	Site	Clinical manifestations				Imaging			Loose bodies	No.	Size (mm)	Treatments (other than synovial chondrectomy)				Remarks
							Swelling	Pain	LJO	Crepitation	CT	MRI	CBCT				Arthrotomy	Condylectomy	Discectomy	Synovectomy	Such as arthroscopy
1	Peng [17]	2009	1	58	F	R	+	+	+	—	+	—	—		—	—	—	—	+	+	
2	Cai [18]	2009	1	21	F	L	—	+	+(28 mm)	+	—	+	—		7	5 × 8	—	—	—	—	Only arthroscopy
3	Balasundaram [19]	2009	1	82	M	L	+	—	+(35 mm)	—	—	—	+		—	—	NL	NL	NL	NL	
4	Zha [20]	2009	1	44	M	L	+	+	+	+	+	+	—		90-95	NL	—	—	—	+	
5	Sato [21]	2010	1	74	F	L	+	+	+(35 mm)	—	+	+	—		180	1-5	—	—	—	—	Arthroscopy
6	Goizueta-Adame [22]	2010	1	15	F	L	—	+	+(29 mm)	—	+	+	—		—	—	+	—	—	—	Osteotomy of the zygomatic arch
7	Goizueta-Adame [22]	2010	1	74	F	L	—	+	+(14 mm)	—	+	+	—		—	—	+	—	—	—	
8	Boffano [23]	2010	1	55	F	R	+	+	—	+	+	—	—		<6	<5	—	—	—	+	
9	Pimenta e Souza [24]	2010	1	28	M	R	+	—	—	+	+	—	—		10	NL	NL	NL	NL	NL	
10	Guarda-Nardini [25]	2010	1	53	F	R	+	+	+(20 mm)	—	X-P only	—	—		2	10-25	NL	NL	NL	NL	
11	Cai [26]	2010	1	48	F	L	—	+	—	+	—	+	—		3	16 × 9	—	—	—	—	Only arthroscopy
12	González-Pérez [27]	2010	1	50	M	L	+	+	+(20 mm)	+	+	+	—		35	2-10	—	+	—	—	
13	Shah [28]	2011	1	25	F	L	—	+	—	—	+	—	—		43	<1.5 × 2.5	—	—	+	+	
14	Mori [29]	2011	1	52	F	L	—	+	-(48 mm)	+	+	—	—		N	VS	—	—	—	—	
15	Lim [30]	2011	1	49	M	R	+	+	+(27 mm)	+	+	—	—		N	VS	—	—	—	—	
16	Chen [31]	2011	1	50	M	L	+	+	+(28 mm)	—	—	—	+		N	VS	+	—	—	—	Arthroscopy
17	Guijarro-Martínez [32]	2011	1	38	F	B	+	—	-(40 mm)	—	—	+	—		N	NL	—	—	—	—	
18	Matsumura [33]	2012	1	46	M	R	—	—	+(24 mm)	+	+	+	—		+	NL	—	+	—	+	Aspiration biopsy
19	Matsumoto [34]	2013	1	34	F	R	—	—	-(42 mm)	+	+	+	+		N	NL	—	—	—	—	Assisted with arthroscopy
20	Coleman [35]	2013	1	63	F	L	+	—	—	—	+	+	—		—	—	—	+	—	—	Aspiration biopsy
21	Valentini [36]	2013	1	60	F	L	+	+	—	—	+	+	—		—	—	—	—	—	—	
22	Sink [37]	2014	1	82	M	L	—	—	-(40 mm)	—	—	+	—		—	—	—	—	—	—	Aspiration biopsy
23	Pau [38]	2014	1	70	M	R	+	+	+	—	+	+	—		—	—	—	+	—	—	Reconstruction of the cranial base
24	Cascone [39]	2914	1	30	M	L	—	+	—	—	—	+	—		11	20-30	—	—	—	+	
25	Pinto [40]	2015	1	54	F	L	+	+	+(20 mm)	—	+	+	—		11	<10	—	—	—	—	
26	Ivask [41]	2015	1	45	F	L	—	+	+(38 mm)	+	+	—	—		N	3-10	+	—	—	—	Arthroscopy
27	Sozzi [42]	2015	1	68	F	R	+	+	+(20 mm)	+	+	+	—		N	VS	—	—	—	—	
28	Yoshitake [43]	2016	1	72	M	R	+	+	+(30 mm)	—	+	+	—		—	—	—	—	—	—	
29	Selvi [44]	2016	1	45	F	R	+	+	+(24 mm)	—	—	+	—		N	NL	+	—	+	—	Arthroscopy, arthroplasty, eminectomy
30	McCaffery [45]	2017	1	34	M	R	+	—	—	—	+	+	—		—	—	—	+	—	—	Osteotomy of the zygomatic arch
31	Khanna [46]	2017	1	43	M	L	+	+	+	—	+	—	—		—	—	—	—	—	—	
32	Khanna [46]	2017	1	35	M	R	+	+	+	—	+	+	—		+	NL	—	—	—	—	Osteotomy of the zygomatic arch
33	Kim [47]	2017	1	26	F	R	—	+	—	—	+	+	—		+	2	—	—	—	—	
34	Kim [47]	2017	1	31	F	L	—	+	-(45 mm)	—	+	+	—		+	7-10	—	—	—	—	
35	Kim [48]	2018	1	50	M	L	+	+	—	—	+	+	—		120	Small	—	—	—	—	
36	Holtmann [49]	2018	1	63	F	R	+	+	—	—	+	+	—		25	14 × 6 VS	—	—	—	—	
37	Matsuda [50]	2019	1	73	F	L	+	+	—	—	+	+	—		44	VS	—	—	—	—	∗1
38	Tang [51]	2019	1	60	F	R	—	+	—	—	+	—	—		—	—	—	—	—	—	
39	Tang [51]	2019	1	38	F	R	+	—	+(25 mm)	—	+	—	+		+	NL	—	—	—	—	
40	Tang [51]	2019	1	30	F	L	+	+	+(28 mm)	—	+	—	+		+	NL	—	—	—	—	
41	Meng [52]	2010	20	44.0 (32-67)	8/12	12/7/1	13	17	14	5	15	13	—		40 > N	VS	8	2	9	—	Arthroscopy: 1
42	Wang [11]	2012	22	45.3 (17-64)	7/15	12/10/0	NL	NL	NL	NL	—	22	—		NL	NL	NL	NL	NL	NL	
43	Cai [53]	2016	33	43.0 (21-62)	7/26	18/15/0	3	20	16/30.5 mm(15-55 mm)	18	4	33	—		N	0.5-16	—	—	—	—	Only arthroscopy: 32 Assisted with arthroscopy: 1
44	Liu [7]	2016	10	47.2 (31-67)	2/8	2/8/0	7	7	7	5	1	—	9		6∗2	NL	—	—	—	—	
45	Bai [54]	2017	36	48.11 (29-65)	11/25	14/22/0	NL	NL	22	9	36	36	—		4∗2	NL	—	—	—	—	Assisted with arthroscopy: 36
46	Brabyn [55]	2018	6	42.0 (33-57)	0/6	0/6/0	2	2	32.3 mm (20-46 mm)	4	5	5	—		6∗2	0.5-3.0	—	—	—	—	
47	Maffia [56]	2019	6	48.6 (30-61)	2/4	2/4/0	5	6	5	3	6	6	—		NL	NL	—	—	—	6	
48	Lee [57]	2019	16	32.68 (19-49)	2/14	7/9/0	—	10	4	8	2	15	—		40-100	0.5-6.5	—	—	—	—	Only arthroscopy: 16
49	Benslama [58]	2019	12	50.5 (43-86)	4/8	3/9/0	4	10	6	2	2	6	—		2-30	VS	—	—	—	—	Assisted with arthroscopy: 1
50	Present case	2020	1	56	F	L	—	+	+(30 mm)	+	+	+	—		386	2-5	+	+	—	—	

## References

[1] Von Haller A. (1765). *Elementa physiologiae corporus humani*.

[2] Al-Kayat A., Bramley P. (1979). A modified pre-auricular approach to the temporomandibular joint and malar arch. *Journal of Oral Surgery*.

[3] Forssell K., Happonen R. P., Forssell H. (1988). Synovial chondromatosis of the temporomandibular joint: report of a case and review of the literature. *International Journal of Oral and Maxillofacial Surgery*.

[4] Lieger O., Zix J., Stauffer-Brauch E. J., Iizuka T. (2007). Synovial chondromatosis of the temporomandibular joint with cranial extension: a case report and literature review. *Journal of Oral and Maxillofacial Surgery*.

[5] Carls F. R., von Hochstetter A., Engelke W., Sailer H. F. (1995). Loose bodies in the temporomandibular joint: the advantages of arthroscopy. *Journal of Cranio-Maxillo-Facial Surgery*.

[6] Ardekian L., Faquin W., Troulis M. J., Kaban L. B., August M. (2005). Synovial chondromatosis of the temporomandibular joint: report and analysis of eleven cases. *Journal of Oral and Maxillofacial Surgery*.

[7] Liu X., Huang Z., Zhu W., Liang P., Tao Q. (2016). Clinical and imaging findings of temporomandibular joint synovial chondromatosis: an analysis of 10 cases and literature review. *Journal of Oral and Maxillofacial Surgery*.

[8] Noyek A. M., Holgate R. C., Fireman S. M., Rosen P., Pritzker K. P. (1977). The radiologic findings in synovial chondromatosis (chondrometaplasia) of the temporomandibular joint. *The Journal of Otolaryngology. Supplement*.

[9] Balliu E., Medina V., Vilanova J. (2007). Synovial chondromatosis of the temporomandibular joint: CT and MRI findings. *Dento Maxillo Facial Radiology*.

[10] Ginaldi S. (1980). Computed tomography feature of synovial osteochondromatosis. *Skeletal Radiology*.

[11] Wang P., Tian Z., Yang J., Yu Q. (2012). Synovial chondromatosis of the temporomandibular joint: MRI findings with pathological comparison. *Dento Maxillo Facial Radiology*.

[12] Von Arx D. P., Simpson M. T., Batman P. (1988). Synovial chondromatosis of the temporomandibular joint. *The British Journal of Oral & Maxillofacial Surgery*.

[13] Yu Q., Yang J., Wang P., Shi H., Luo J. (2004). CT features of synovial chondromatosis in the temporomandibular joint. *Oral Surgery, Oral Medicine, Oral Pathology, Oral Radiology, and Endodontics*.

[14] Milgram J. W. (1977). Synovial osteochondromatosis: a histopathological study of thirty cases. *The Journal of Bone and Joint Surgery. American Volume*.

[15] Milgram J. W. (1977). The classification of loose bodies in human joints. *Clinical Orthopaedics and Related Research*.

[16] Gerard Y., Shall A., Ameil M. (1993). Synovial osteochondromatosis. Therapeutic indications based on a histological classification. *Chirurgie; memoires de l'Academie de chirurgie*.

[17] Peng L. W., Yan D. M., Wang Y. G., Li Y. D. (2009). Synovial chondromatosis of the temporomandibular joint: a case report with bilateral occurrence. *Journal of Oral and Maxillofacial Surgery*.

[18] Cai X. Y., Yang C., Chen M. J., Yun B. (2009). Simultaneous pigmented villonodular synovitis and synovial chondromatosis of the temporomandibular joint: case report. *International Journal of Oral and Maxillofacial Surgery*.

[19] Balasundaram A., Geist J. R., Gordon S. C., Klasser G. D. (2009). Radiographic diagnosis of synovial chondromatosis of the temporomandibular joint: a case report. *Journal of the Canadian Dental Association*.

[20] Zha W., Zhao Y. F., Liu Y., Jiang L. (2009). A case of synovial chondromatosis of the temporomandibular joint secondary to preauricular trauma. *International Journal of Oral and Maxillofacial Surgery*.

[21] Sato J., Notani K. I., Goto J., Shindoh M., Kitagawa Y. (2010). Synovial chondromatosis of the temporomandibular joint accompanied by loose bodies in both the superior and inferior joint compartments: case report. *International Journal of Oral and Maxillofacial Surgery*.

[22] Goizueta-Adame C. C., González-García R. (2010). Synovial chondromatosis of the temporomandibular joint: report of 2 patients whose joints were reconstructed with costochondral graft and alloplastic prosthesis. *The British Journal of Oral & Maxillofacial Surgery*.

[23] Boffano P., Viterbo S., Bosco G. F. (2010). Diagnosis and surgical management of synovial chondromatosis of the temporomandibular joint. *The Journal of Craniofacial Surgery*.

[24] Souza D. P., Loureiro C. C., Falchet P. F., Leandro L. F., Raitz R. (2010). Synovial chondromatosis of the temporomandibular joint: an asymptomatic case report and literature review. *Cranio*.

[25] Guarda-Nardini L., Piccotti F., Ferronato G., Manfredini D. (2010). Synovial chondromatosis of the temporomandibular joint: a case description with systematic literature review. *International Journal of Oral and Maxillofacial Surgery*.

[26] Cai X. Y., Yang C., Chen M. J., Jiang B., Wang B. L. (2010). Arthroscopically guided removal of large solitary synovial chondromatosis from the temporomandibular joint. *International Journal of Oral and Maxillofacial Surgery*.

[27] González-Pérez L. M., Congregado-Córdoba J., Salinas-Martín M. V. (2011). Temporomandibular joint synovial chondromatosis with a traumatic etiology. *International Journal of Oral and Maxillofacial Surgery*.

[28] Shah S. B., Ramanojam S., Gadre P. K., Gadre K. S. (2011). Synovial chondromatosis of temporomandibular joint: journey through 25 decades and a case report. *Journal of Oral and Maxillofacial Surgery*.

[29] Mori Y., Kakudo K., Gotoh M. (2011). A case of synovial chondromatosis of the temporomandibular joint followed for 17 years. *Oral Surgery, Oral Medicine, Oral Pathology, Oral Radiology, and Endodontics*.

[30] Lim S. W., Jeon S. J., Choi S. S., Choi K. H. (2011). Synovial chondromatosis in the temporomandibular joint: a case with typical imaging features and pathological findings. *The British Journal of Radiology*.

[31] Chen M. J., Yang C., Zhang X. H., Qiu Y. T. (2011). Synovial chondromatosis originally arising in the lower compartment of temporomandibular joint: a case report and literature review. *Journal of Cranio-Maxillo-Facial Surgery*.

[32] Guijarro-Martínez R., Puche Torres M., Marqués Mateo M. (2011). Bilateral synovial chondromatosis of the temporomandibular joint. *Journal of Cranio-Maxillo-Facial Surgery*.

[33] Matsumura Y., Nomura J., Nakanishi K., Yanase S., Kato H., Tagawa T. (2012). Synovial chondromatosis of the temporomandibular joint with calcium pyrophosphate dihydrate crystal deposition disease (pseudogout). *Dento Maxillo Facial Radiology*.

[34] Matsumoto K., Sato T., Iwanari S. (2013). The use of arthrography in the diagnosis of temporomandibular joint synovial chondromatosis. *Dento Maxillo Facial Radiology*.

[35] Coleman H., Chandraratnam E., Morgan G., Gomes L., Bonar F. (2013). Synovial chondrosarcoma arising in synovial chondromatosis of the temporomandibular joint. *Head and Neck Pathology*.

[36] Valentini V., Arangio P., Egidi S. (2013). Diagnosis and treatment of synovial chondromatosis of the TMJ: a clinical case. *Ann Stomatol*.

[37] Sink J., Bell B., Mesa H. (2014). Synovial chondromatosis of the temporomandibular joint: clinical, cytologic, histologic, radiologic, therapeutic aspects, and differential diagnosis of an uncommon lesion. *Oral Surgery, Oral Medicine, Oral Pathology, Oral Radiology*.

[38] Pau M., Bicsák Á., Reinbacher K. E., Feichtinger M., Kärcher H. (2014). Surgical treatment of synovial chondromatosis of the temporomandibular joint with erosion of the skull base: a case report and review of the literature. *International Journal of Oral and Maxillofacial Surgery*.

[39] Cascone P., Gennaro P., Gabriele G. (2014). Temporomandibular synovial chondromatosis with numerous nodules. *The Journal of Craniofacial Surgery*.

[40] Pinto A. A., Ferreira e Costa R., de Sousa S. F., Chagas M. R. P., do Carmo M. A. V., de Lacerda J. C. T. (2015). Synovial chondromatosis of the temporomandibular joint successfully treated by surgery. *Head and Neck Pathology*.

[41] Ivask O., Leibur E., Voog-Oras Ü. (2015). Synovial chondromatosis in the temporomandibular joint: case report with review of the literature. *Stomatologija*.

[42] Sozzi D., Bocchialini G., Novelli G., Valente M. G., Moltrasio F., Bozzetti A. (2015). A rare case of synovial chondromatosis of the inferior TMJ compartment. Diagnosis and treatment aspect. *Annali di stomatologia*.

[43] Yoshitake H., Kayamori K., Nakamura R., Wake S., Harada K. (2015). Pseudotumor in the temporomandibular joint: a case report. *International Journal of Surgery Case Reports*.

[44] Selvi F., Messina J., Faquin W. C., Keith D. A. (2016). Relapsing polychondritis concomitant with synovial chondromatosis of the temporomandibular joint. *Journal of Oral and Maxillofacial Surgery*.

[45] McCaffery C., Dodd M., Bekiroglu F., Twohig E. (2017). Synovial chondromatosis of the temporomandibular joint with extension into the middle cranial fossa and internal carotid canal. *International Journal of Oral and Maxillofacial Surgery*.

[46] Khanna J. N., Ramaswami R. (2017). Synovial chondromatosis of the temporomandibular joint with intracranial extension--report of two cases. *International Journal of Oral and Maxillofacial Surgery*.

[47] Kim D. H., Lee E. H., Cho E. S. (2017). Temporomandibular joint synovial chondromatosis extending to the temporal bone: a report of two cases. *Journal of the Korean Association of Oral and Maxillofacial Surgeons*.

[48] Kim H. S., Lee W., Choi J. W., Han W. J., Kim E. K. (2018). Temporomandibular joint synovial chondromatosis accompanying temporal bone proliferation: a case report. *Imaging Sci Dent*.

[49] Holtmann H., Böttinger T., Kübler N. R. (2018). Intra- and extracapsular synovial chondromatosis of the temporomandibular joint: rare case and review of the literature. *SAGE open medical case reports*.

[50] Matsuda S., Yoshimura H., Sano K. (2019). Application of a real-time 3-dimensional navigation system for treatment of synovial chondromatosis of the temporomandibular joint: a case report. *Medicine (Baltimore)*.

[51] Tang B., Wang K., Wang H., Zheng G. (2019). Radiological features of synovial chondromatosis affecting the temporomandibular joint: report of three cases. *Oral Radiology*.

[52] Meng J., Guo C., Yi B., Zhao Y., Luo H., Ma X. (2010). Clinical and radiologic findings of synovial chondromatosis affecting the temporomandibular joint. *Oral Surgery, Oral Medicine, Oral Pathology, Oral Radiology, and Endodontics*.

[53] Cai X. Y., Yang C., Chen M. J. (2012). Arthroscopic management for synovial chondromatosis of the temporomandibular joint: a retrospective review of 33 cases. *Journal of Oral and Maxillofacial Surgery*.

[54] Bai G., Yang C., Qiu Y., Chen M. (2017). Open surgery assisted with arthroscopy to treat synovial chondromatosis of the temporomandibular joint. *International Journal of Oral and Maxillofacial Surgery*.

[55] Brabyn P. J., Capote A., Muñoz-Guerra M. F., Zylberberg I., Rodríguez-Campo F. J., Naval-Gías L. (2018). Arthroscopic management of synovial chondromatosis of the temporomandibular joint. Case series and systematic review. *J Maxillofac Oral Surg*.

[56] Maffia F., Vellone V., De Quarto C., Runci Anastasi M., Cascone P. (2019). Synovial chondromatosis of the temporomandibular joint with glenoid fossa erosion: disk preservation for spontaneous anatomical recovery. *Journal of Cranio-Maxillo-Facial Surgery*.

[57] Lee L. M., Zhu Y. M., Zhang D. D., Deng Y. Q., Gu Y. (2019). Synovial chondromatosis of the temporomandibular joint: a clinical and arthroscopic study of 16 cases. *Journal of Cranio-Maxillo-Facial Surgery*.

[58] Benslama L., Schouman T., Toure S., Chardain J., Goudot P. (2019). Synovial chondromatosis of the temporomandibular joint: report and analysis of 12 cases. *J Stomatol Oral Maxillofac Surg*.

[59] Ishii J., Kino K., Kobayashi J., Amagasa T. (2003). Synovial chondromatosis of the temporomandibular joint: long-term postoperative follow-up of the residual calcification. *Journal of Medical and Dental Sciences*.

[60] Han Z. X., Chen M. J., Yang C., Dong M. J. (2017). Recurrent synovial chondromatosis of the temporomandibular joint: report of two cases. *The British Journal of Oral & Maxillofacial Surgery*.

[61] Deboise A., Roche Y. (1991). Synovial chondromatosis of the temporomandibular joint possibly secondary to trauma: a case report. *International Journal of Oral and Maxillofacial Surgery*.

[62] Li Y., El Mozen L. A., Cai H. (2015). Transforming growth factor beta 3 involved in the pathogenesis of synovial chondromatosis of temporomandibular joint. *Scientific Reports*.

[63] Sato J., Segami N., Suzuki T., Yoshitake Y., Nishikawa K. (2002). The expression of fibroblast growth factor-2 and fibroblast growth factor receptor-1 in chondrocytes in synovial chondromatosis of the temporomandibular joint. Report of two cases. *International Journal of Oral and Maxillofacial Surgery*.

[64] Ikebe T., Nakayama E., Shinohara M., Takeuchi H., Takenoshita Y. (1998). Synovial chondromatosis of the temporomandibular joint: the effect of interleukin-1 on loose-body-derived cells. *Oral Surgery, Oral Medicine, Oral Pathology, Oral Radiology, and Endodontics*.

[65] Suzuki T., Segami N., Nishimura M., Hattori H., Nojima T. (2000). Analysis of 70Kd heat shock protein expression in patients with internal derangement of the temporomandibular joint. *International Journal of Oral and Maxillofacial Surgery*.

[66] Anract P., Katabi M., Forest M., Benoit J., Witvoët J., Tomeno B. (1996). Synovial chondromatosis and chondrosarcoma. A study of the relationship between these two diseases. *Revue de Chirurgie Orthopédique et Réparatrice de l'Appareil Moteur*.

[67] Morales-Ducret J., Wayner E., Elices M. J., Alvaro-Gracia J. M., Zvaifler N. J., Firestein G. S. (1992). Alpha 4/beta 1 integrin (VLA-4) ligands in arthritis. Vascular cell adhesion molecule-1 expression in synovium and on fibroblast-like synoviocytes. *Journal of Immunology*.

[68] Fujita S., Yoshida H., Tojyo I., Wada T., Murakami K., Iizuka T. (2004). Synovial chondromatosis of the temporomandibular joint: clinical and immunohistopathological considerations. *The British Journal of Oral & Maxillofacial Surgery*.

[69] Kenan S., Abdelwahab I. F., Klein M. J., Lewis M. M. (1993). Case report 817: synovial chondrosarcoma secondary to synovial chondromatosis. *Skeletal Radiology*.

[70] Campbell D. I., De Silva R. K., De Silva H., Sinon S. H., Rich A. M. (2011). Temporomandibular joint synovial chondromatosis with intracranial extension: a review and observations of patient observed for 4 years. *Journal of Oral and Maxillofacial Surgery*.

